# Trends in Labor After Cesarean Delivery Access in the US

**DOI:** 10.1001/jamanetworkopen.2025.26224

**Published:** 2025-08-08

**Authors:** Brittany L. Ranchoff, Kimberley H. Geissler, Sarah L. Goff, Elizabeth R. Bertone-Johnson, Mary T. Paterno, Laura B. Attanasio

**Affiliations:** 1 Department of Population Medicine, Harvard Medical School and Harvard Pilgrim Health Care Institute, Boston, Massachusetts; 2 Department of Health Promotion and Policy, School of Public Health and Health Sciences, University of Massachusetts Amherst, Amherst; 3 Department of Healthcare Delivery and Population Sciences, University of Massachusetts Chan Medical School—Baystate, Springfield; 4 Department of Biostatistics and Epidemiology, School of Public Health and Health Sciences, University of Massachusetts Amherst, Amherst; 5 Department of Obstetrics and Gynecology, University of Massachusetts Chan Medical School—Baystate, Springfield

## Abstract

This cross-sectional study examines county-level trends in labor after cesarean delivery access across the US from 2016 to 2021.

## Introduction

Despite the potential benefits of vaginal birth after cesarean (VBAC) delivery, access to labor after cesarean (LAC) delivery is limited for many individuals with a prior cesarean birth, as many clinicians and hospitals do not offer LAC services.^1,2,3^ Although clinical practice guidelines have become somewhat more encouraging of VBAC, the extent to which LAC services are available nationally in recent years is unknown. We examined county-level trends in LAC access across the continental US from 2016 to 2021.

## Methods

This cross-sectional study used data from the Natality Restricted-Use Data Files (2016 to 2021), which captures all US births. Our outcome was a binary measure of LAC access in each county-year, defined as at least 1 hospital offering LAC. We adapted prior methods for determining hospital-level LAC access to the county-level based on rates of LAC, LAC ending in VBAC, and prior cesarean birth, as well as an estimated expected LAC count.^3^ Counties with near zero LAC and/or lower than expected LAC counts were considered to not have LAC access. We descriptively examined LAC access by year and other factors, using χ^2^ tests and *t* tests. We then used a generalized estimating equation model to conduct multivariate analysis for county-level LAC access by year, controlling for other county characteristics. Finally, we repeated the analysis limited to counties with an obstetric hospital, because approximately 50% of counties do not have an obstetric hospital. This cross-sectional study was reviewed and approved by the University of Massachusetts institutional review board and Strengthening the Reporting of Observational Studies in Epidemiology (STROBE) reporting guideline.

## Results

The final analytic sample included 18 648 county-years (3108 counties) from 2016 to 2021. The Figure A and B depict the geographic distribution of LAC access in 2016 (first year in study) and 2021 (last year in study). Counties with LAC access were more prevalent in the Northeast and Western regions.

**Figure.**
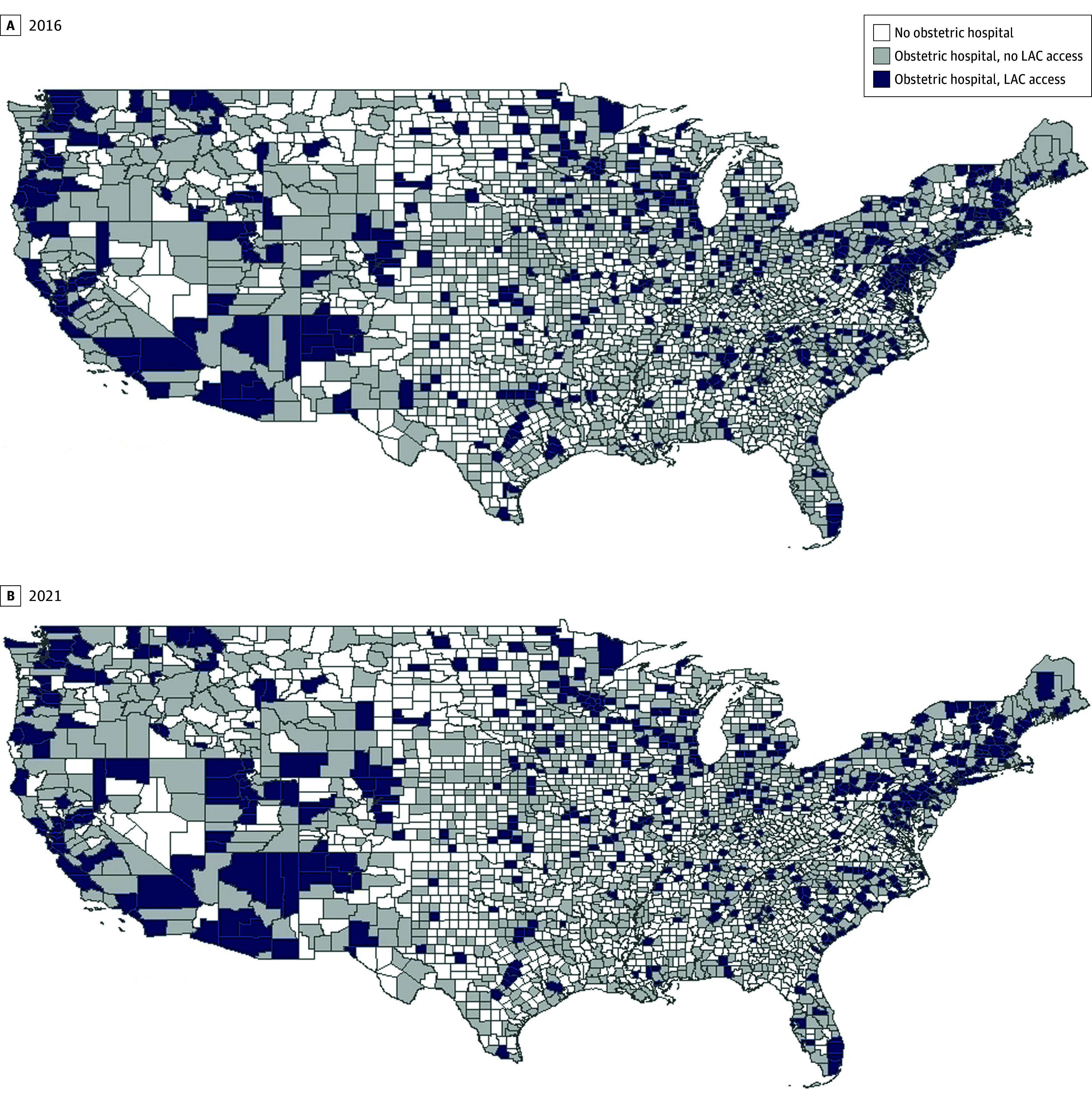
County-Level Labor After Cesarean (LAC) Access

Throughout the study, 2957 county-years (15.9%) had LAC access (Table). Nearly a third of metro counties had LAC access (2123 county-years [30.5%]), compared with 458 county-years in metro-adjacent counties (7.4%) and 376 county-years in metro-nonadjacent counties (6.8%). Among counties with hospital-based obstetric services only, 2957 county-years (30.9%) had LAC access. LAC access did not vary across years in bivariate or multivariate analyses. In the subgroup analysis limiting to counties with an obstetric hospital, results were similar in magnitude, direction, and significance as the main analysis.

**Table.** Sample Characteristics by County-Level LAC Access and the Adjusted Generalized Estimating Equation Model for Year and LAC-Offering County

County characteristics	County-years, No. (%)^a^			*P* value	County-level LAC access, aOR (95% CI)^b^	*P* value
	Overall	County-level LAC access				
		No	Yes			
County-level LAC access	18 648 (100.00)	15 691 (84.1)	2957 (15.9)			
Year						
2016	3108 (16.7)	2602 (83.7)	506 (16.3)	.98	1 [Reference]	NA
2017	3108 (16.7)	2619 (84.3)	489 (15.7)		0.92 (0.83-1.01)	.08
2018	3108 (16.7)	2609 (83.9)	499 (16.1)		0.94 (0.84-1.05)	.27
2019	3108 (16.7)	2625 (84.5)	483 (15.5)		0.89 (0.79-1.01)	.06
2020	3108 (16.7)	2617 (84.2)	491 (15.8)		0.89 (0.78-1.02)	.09
2021	3108 (16.7)	2619 (84.3)	489 (15.7)		0.89 (0.77-1.01)	.08
Rurality						
Metropolitan county	6960 (37.3)	4837 (69.5)	2123 (30.5)	<.001	1 [Reference]	NA
Nonmetropolitan county adjacent to a metro area	6156 (33.0)	5698 (92.6)	458 (7.4)		0.27 (0.21-0.35)	<.001
Nonmetropolitan county nonadjacent to a metro area	5532 (29.7)	5156 (93.2)	376 (6.8)		0.22 (0.16-0.31)	<.001
County with lowest-quartile median income						
No	8298 (44.5)	6235 (75.1)	2063 (24.9)	<.001	1 [Reference]	NA
Yes	10 350 (55.5)	9456 (91.4)	894 (8.6)		0.96 (0.81-1.13)	.62
% Of adult population who are insured by Medicaid, mean (SD)	14.69 (7.5)	14.92 (7.7)	13.5 (6.4)	<.001	0.94 (0.93-0.96)	<.001
% Of population who are Black or African American, mean (SD)	10.02 (14.7)	9.80 (15.0)	11.2 (13.0)	<.001	1.04 (1.03-1.05)	<.001
% Of population who are Hispanic or Latino, mean (SD)	9.39 (13.9)	8.91 (13.9)	12.0 (13.3)	<.001	1.03 (1.02-1.04)	<.001
Uninsured rate, mean (SD)	10.32 (5.1)	10.60 (5.2)	8.8 (4.3)	<.001	0.97 (0.94-0.99)	.01
No. of obstetrician-gynecologists per 100 000 adult reproductive-aged female people, mean (SD)	25.61 (34.8)	22.1 (32.4)	62.8 (42.8)	<.001	1.02 (1.01-1.02)	<.001
No. of midwives per 100 000 adult reproductive-aged female people, mean (SD)	7.13 (17.5)	5.27 (16.1)	17.0 (21.0)	<.001	1.02 (1.01-1.02)	<.001
Region						
Northeast	1302 (7.0)	776 (59.6)	526 (40.4)	<.001	NA	NA
Midwest	6330 (33.9)	5413 (85.5)	917 (14.5)		NA	NA
South	8532 (45.8)	7619 (89.3)	913 (10.7)		NA	NA
West	2484 (13.3)	1883 (75.8)	601 (24.2)		NA	NA
County with an obstetric hospital						
No	9077 (48.7)	9077 (100.0)	NA	<.001	NA	NA
Yes	9571 (51.3)	6614 (69.1)	2957 (30.9)		NA	NA

Abbreviations: aOR, adjusted odds ratio; LAC, labor after cesarean; NA, not applicable.

^a^ A county-year was defined as 1 year based on birth data. Each county had 6 observation periods in the dataset from 2016 to 2021; thus, each county was represented by 6 county-years.

^b^ Adjusted model controls for number of obstetrician-gynecologists per 100 000 adult reproductive-aged female people; number of midwives per 100 000 adult reproductive-aged female people; uninsured rate; percentage of adult population who are insured by Medicaid; percentage of population who are Black or African American; percentage of population who are Hispanic or Latino; county with lowest-quartile median income; and rurality. Analysis also adjusts for state fixed effects. Standard errors are clustered at the county. District of Columbia (DC) was dropped from the model. N = 18 642 county-years.

## Discussion

We found fewer than 16% of counties offered LAC throughout the study, with extremely limited access to LAC in some regions. Prior studies have examined LAC access within specific states, but no recent studies have assessed county-level LAC access nationally.^1,2,3^ We found limited but stable LAC availability overall and when limiting to counties with obstetric hospitals, despite increases in VBAC rates between 2011 and 2021.^4^ Our findings confirm the difficulty individuals have expressed in accessing LAC.^5^ Although the American College of Obstetricians and Gynecologists states LAC is a reasonable option for many individuals with a prior cesarean delivery, many birthing people may not be able to access LAC due to a lack of local availability. Other barriers, such as insurance and transportation limitations, may limit LAC access even for individuals in counties where LAC is available.

Study limitations include the quality of birth certificate items which could lead to potential misclassification in defining LAC-offering counties.^6^ Despite small increases in the US VBAC rate in recent years,^4^ our results indicate LAC access continues to be limited, suggesting LAC and VBAC may have become more concentrated among a smaller set of hospitals or clinicians. Future research should investigate the consequences of limited LAC access on perinatal care experiences and outcomes for the substantial proportion of individuals with a prior cesarean delivery.

## Notes

Supplement.
Data Sharing Statement
