# Supplementary material for: Trends in Labor After Cesarean Delivery Access in the US
Source: JAMA Netw Open. 2025 Aug 8;8(8):e2526224. doi: 10.1001/jamanetworkopen.2025.26224 (PMC12334954; doi:10.1001/jamanetworkopen.2025.26224)
Supplement: Supplement. — Data Sharing Statement [file jamanetwopen-e2526224-s001.pdf]

Data Sharing Statement

Ranchoff. Trends in Labor After Cesarean Delivery Access in the US. *JAMA Netw Open*. Published August 08, 2025. doi:10.1001/jamanetworkopen.2025.26224

Data
Data available: No
